# Assessment of New Inflammatory Indexes in Systemic Sclerosis with Interstitial Lung Disease

**DOI:** 10.5152/eurasianjmed.2025.251103

**Published:** 2025-10-23

**Authors:** Ecesoy Volkan, Ecesoy Hilal

**Affiliations:** 1 Department of Biochemistry, Karamanoğlu Mehmetbey University Faculty of Medicine, Karaman, Türkiye; 2 Division of Rheumatology, Department of Physical Therapy and Rehabilitation, Karamanoğlu Mehmetbey University Faculty of Medicine, Karaman, Türkiye

**Keywords:** Disease activity, inflammatory markers, interstitial lung disease, systemic sclerosis

## Abstract

**Background:**

Systemic sclerosis (SSc) is a chronic autoimmune inflammatory disease. The most significant and prevalent complication of SSc is interstitial lung disease (ILD). It is difficult to monitor disease activity. In outpatient clinic conditions, C-reactive protein remains nonspecific and specific methods such as the European Scleroderma Trials and Research group take a long time. This study aimed to evaluate the effectiveness of new inflammation markers obtained from blood parameters, which is a rapid and inexpensive method, in SSc patients with ILD.

**Methods:**

A total of 53 patients with SSc and 54 healthy subjects were included in this retrospective cross-sectional study.

**Results:**

The neutrophil-to-lymphocyte ratio (NLR), platelet-to-lymphocyte ratio (PLR), systemic inflammatory index (SII), systemic inflammatory response index (SIRI), and pan-immune-inflammation values (PIV) were found to be higher in the patient group than in the control group. The lymphocyte-to-monocyte ratio (LMR) value was significantly lower in the patient group. Besides, these patients with ILD had significantly higher NLR, PLR, SII, and PIV values. The SIRI values were also high, but not statistically significant. The LMR value was found to be significantly lower. The SIRI values with 66% sensitivity and 66.7% specificity for SSc patients, and SII values with 75% sensitivity and 74.7% specificity for SSc patients with ILD can indicate disease activity.

**Conclusion:**

The novel indices may prove superior to rudimentary indices by virtue of their capacity to more accurately reflect inflammatory activity in inflammatory connective tissue diseases, particularly those accompanied by fibrotic lung involvement. These indices have the potential to serve as a cost-effective predictive instrument.

**Main Points**

- There is no definitive disease-specific biomarker that indicates disease activity in systemic sclerosis (SSc).
- Ratios obtained from the proportion of complete blood parameters can provide insight into the severity of ongoing inflammation.
- New biomarkers such as systemic inflammatory index, systemic inflammatory response index, and pan-immune-inflammation value are more stable than individual blood count results and can be calculated in as little as a few minutes.
- New biomarkers are superior to simple indices in determining activity in patients with SSc, especially those with interstitial lung disease, and can be used as a cost-effective predictive tool.

## Introduction

Systemic sclerosis (SSc) is a chronic autoimmune disorder characterized by persistent inflammation, with its underlying etiology remaining unidentified. It is characterized by widespread fibrosis and vascular abnormalities affecting both the internal organs and skin.^1^ This condition is mostly observed in women of childbearing age, typically between the ages of 30 and 55. Despite the rarity of the condition, it can result in significant involvement of vital internal organs, thereby posing a substantial risk to life.^2^ The most significant and prevalent of these complications is that of lung involvement. Pulmonary arterial hypertension (PAH), one of the pulmonary involvements, may be a direct consequence of remodeling of pre-capillary pulmonary arterioles or may occur due to left heart dysfunction secondary to interstitial lung disease (ILD). Another form of lung involvement seen in SSc is ILD, with a pattern called nonspecific interstitial pneumonitis with ground-glass opacities.^3^ The frequency of ILD varies according to the diagnostic method used, but it affects approximately 50% of SSc patients.^4^ Interstitial lung disease has recently been identified as the primary cause of mortality associated with SSc, accounting for 17%-35% of all SSc-related deaths.^5^

Erythrocyte sedimentation rate (ESR) and serum C-reactive protein (CRP) are commonly used inflammatory biomarkers to monitor disease activity in rheumatic diseases, but they are not very sensitive or specific. Furthermore, it is not possible to evaluate scores such as the European Scleroderma Trials and Research group SSc activity score or the Medsger SSc severity scale, which are used to assess disease activity in SSc, in outpatient clinic conditions over time. Consequently, there is a need for novel biomarkers that more accurately reflect disease activity. Previous studies have explored the neutrophil-to-lymphocyte ratio (NLR), platelet-to-lymphocyte ratio (PLR), and lymphocyte-to-monocyte ratio (LMR) as potential inflammation-related indicators for predicting disease activity in various rheumatic disorders. These ratios are obtained by dividing simple hematology parameters by each other.^6^^,^^7^

Recently, the newly developed biomarkers systemic inflammatory index (SII), systemic inflammatory response index (SIRI) and pan-immune-inflammation value (PIV) have been investigated in many inflammation-related diseases. Research has demonstrated that these biomarkers can serve as prognostic indicators, particularly in solid organ cancers, idiopathic pulmonary fibrosis (IPF), and inflammatory conditions including rheumatoid arthritis (RA) and psoriasis.^6^^-^^9^

This study compared classical inflammation biomarkers obtained from ESR, CRP, and blood count data with newly derived biomarkers from blood count data in patients with SSc disease. The objective was to assess whether these findings could be utilized to track disease progression in patients with ILD.

## Materials and Methods

This case-control study included 53 patients with SSc who were classified according to the classification criteria for SSc of the American College of Rheumatology/European League against Rheumatism^10^ and 54 healthy controls who were admitted to the Rheumatology outpatient clinic of Karamanoğlu Mehmetbey University Training and Research Hospital. Ethical approval was obtained from Ethics Committee of Karamanoğlu Mehmetbey University before the study began (Approval no: E-11095095-050.04-258232, Date: May 12, 2025). After obtaining ethics committee approval, the study data were scanned retrospectively from the hospital’s automation system, covering a 1-year period between May 2024 and May 2025. The control group consisted of age- and gender-matched healthy hospital employees who had no history of chronic disease and were not using medication.

The principles belonging to the Declaration of Helsinki were adhered to, and all participants provided informed consent. Patients under 18 years of age, patients with a systemic infection or an oncological, inflammatory/autoimmune, or chronic disease other than SSc were not included in the study. Demographic data, hematology results, and biochemical parameters for both the group of patients with SSc disease and the control group were obtained from the hospital’s automated system.

Whole blood count parameters of all participants, including white blood cell count, neutrophils, lymphocytes, monocytes, and platelets were recorded. The formulas were calculated as follows:

Neutrophil-to-lymphocyte ratio (NLR: neutrophils/lymphocytes),

Lymphocyte-to-monocyte ratio (LMR: lymphocytes / monocytes),

Platelet-to-lymphocyte ratio (PLR: platelet/lymphocyte),

Systemic inflammatory index (SII: neutrophils × platelets/lymphocytes),

Systemic inflammatory response index (SIRI: neutrophils × monocytes/lymphocytes),

Pan-immune-inflammation value (PIV: neutrophils × platelets × monocytes/lymphocytes).

The presence of ILD in patients was assessed based on both their medical history and thoracic computed tomography scan results. The control group had no respiratory complaints and therefore no chest radiographs.

### Statistics

The data were analyzed using IBM SPSS V23 (IBM SPSS Corp.; Armonk, NY, USA). The normality of the distribution was assessed using the Shapiro–Wilk and Kolmogorov–Smirnov tests. An independent 2-sample *t*-test was used to compare normally distributed data between paired groups, while the Mann–Whitney *U*-test was applied for non-normally distributed data. The results of the analyses are presented as the mean ± standard deviation and the median (minimum–maximum) for quantitative data. A *P*-value below .05 was considered statistically significant. The optimal cutoff values for SII, SIRI, and PIV were calculated using the receiver operating characteristic (ROC) curve. The correlation between the parameters was determined using the Spearman rank-order correlation test.

## Results

For this study, the data of 53 patients with SSc and 54 healthy individuals who were matched for age and sex were retrospectively evaluated. The demographic characteristics of both groups are shown in Table 1. There was no significant difference between the 2 groups with respect to age and sex. Skin involvement was present in 81% of the patients and 62% complained of Raynaud’s phenomenon. 53% of patients had articular complaints. A small proportion of patients had gastrointestinal involvement (7.5%), while a considerable number had pulmonary involvement (ILD—45%, PAH—15%). Hydroxychloroquine only (HQ) was used in 28% of patients, immunosuppressive only (IMMSUP) in 19% (azathioprine or mycophenolate mofetil), while 53% received both HQ and IMMSUP. No patient received steroid treatment.

Seventeen of the 53 patients included in the study had a history of digital ulcers. However, when their medical histories were reviewed at the time the study data were collected, it was found that none of them had an active digital ulcer at that time.

When the hematological data and inflammatory indices of the patient and control groups were compared (see Table 2), no significant differences were found in the white blood cell (WBC), neutrophil, monocyte, platelet, CRP values, and ESR. However, lymphocyte values were significantly lower in the patient group than in the control group (*P* = .001). The SII, SIRI, and PIV values, which are relatively new biomarkers compared to the NLR and PLR values, were found to be higher in the patient group than in the control group (*P* = .001, *P* = .01, *P* = .012, *P* = .003 and *P* = .013, respectively). The LMR value was significantly lower in the patient group (*P* < .001).

When patients with and without ILD were compared (see Table 3), it was found that those with ILD had significantly higher CRP, NLR, PLR, SII, and PIV values. The SIRI values were also high, but not statistically significant. The LMR value was found to be significantly lower.

Notable differences in age and lymphocyte count were observed when patients with SSc and ILD were compared to the control group. The ILD group was older and had a lower lymphocyte count. The CRP, NLR, PLR, SIRI, and SII values were all statistically significantly higher in patients with ILD. The LMR value was found to be significantly lower (see Table 3).

Compared with the control group, SIRI values were significantly higher and LMR values significantly lower in patients with SSc without ILD (see Table 3).

The treatments received by patients depended on their level of involvement. When the effects of these differences on new inflammation markers were evaluated, it was found that treatment had no impact on the results (see Table 4).

**Figure 1.**
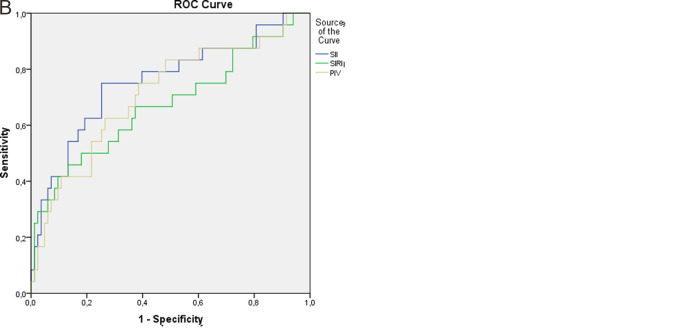
A: The ROC curve of SII, PIV, and SIRI parameters for systemic sclerosis. B: The ROC curve of SII, PIV, and SIRI parameters for systemic sclerosis with ILD.

The area under the curve (AUC) for the SII score in patients with SSc was 0.642. A cutoff value of 533.7 was identified for predicting SSc disease activation using the SII, with a sensitivity of 58.5% and a specificity of 61.1%. Similarly, SIRI values above 0.89 (AUC: 0.665), with 66% sensitivity and 66.7% specificity, and PIV values above 228.07 (AUC: 0.640), with 60.4% sensitivity and 61.1% specificity, are considered risk factors for active SSc (Table 5 and Figure 1A).

For SSc patients with ILD, the SII cutoff value was determined to be 642.97, exhibiting 75% sensitivity and 74.7% specificity (AUC: 0.756). In a similar vein, SIRI values exceeding 0.96 with 62.5% sensitivity and 63.9% specificity (AUC: 0.673) and PIV values greater than 253.36 with 66.7% sensitivity and 65.1% specificity (AUC: 0.710) are recognized as risk factors for SSc patients with ILD (Table 5 and Figure 1B).

As neutrophil, lymphocyte, monocyte, and platelet values are utilized in the calculation of novel indices derived from hemogram parameters, it is anticipated that the indices employed in the study will demonstrate a high correlation with each other. For this reason, correlation analyses were performed between the indices and CRP and ESR. The findings of this study demonstrated that SII and PIV exhibited moderate correlations with ESR and CRP, while SIRI demonstrated a low correlation with ESR and a moderate correlation with CRP. Furthermore, NLR and PLR were found to have moderate correlations with CRP. All statistically significant correlations were found to be positive (see Table 6).

## Discussion

The pathophysiology of SSc has not yet been fully established. In the initial stages of the disease, inflammation is the main pathological process driving clinical symptoms. The catastrophic effects of this inflammation lead to irreversible organ damage in the later stages of the disease. As organ involvement in SSc typically occurs within the first 2-5 years, identifying the factors that determine the severity of inflammation during this period is crucial for predicting the disease’s prognosis.^3^

The ILD and/or PAH, the most common manifestations of the disease, affect approximately 40%-60% of patients with SSc. Lung involvement is responsible for approximately 35% of all SSc-associated deaths.^11^

Thus, in the initial phases, identification of patients at risk for severe organ involvement and life-threatening complications is crucial, as is closely monitoring them and starting effective immunosuppressive treatment promptly. This improves patients’ quality of life and survival.

Despite ongoing intensive research into SSc, there is still no clear, definitive biomarker of disease activity in SSc. Multiple studies have shown that at least 240 pathways and various dysregulated proteins are involved in the pathogenesis of SSc, thus complicating the identification of biomarkers in this disease.^11^

The CRP levels and ESR are currently used to monitor patients with SSc. These tests may indicate disease activity, especially in the early stages of the disease.^12^ However, it should be noted that these values are not specific to the disease under investigation and are also affected by conditions such as infection, pregnancy, and obesity, among others.

Leukocyte subtypes, such as neutrophils, lymphocytes, and monocytes, are used as nonspecific cellular markers of systemic inflammation to diagnose, monitor, and evaluate activation in inflammatory connective tissue diseases. The cellular components of blood and their ratios are known to provide insight into the severity of ongoing inflammation.^13^

Neutrophils contribute to the initiation and progression of the inflammatory response by releasing proinflammatory cytokines. High neutrophil activity, together with increased lymphocyte apoptosis, indicates uncontrolled systemic inflammation, which is why NLR reflects a proinflammatory state. Furthermore, neutrophils are known to induce endothelial cell apoptosis, which is considered one of the initial pathogenic events in SSc.^14^

Recent studies have demonstrated that parameters such as the NLR, PLR, and LMR values, which are obtained from the cellular components of blood, can reflect disease activity in systemic autoimmune disorders such as systemic lupus erythematosus (SLE), SSc, RA, and ankylosing spondylitis.^3^^,^^15^^,^^16^ Similar to studies on connective tissue diseases in the current literature.^17^ This study found a significant increase in NLR and PLR values, and a significant decrease in LMR values, in patients with SSc.^18^^,^^19^

Additionally, although the PLR and NLR values were lowest in the control group, they gradually increased in the SSc-nonILD and SSc-ILD groups, respectively. The LMR was lowest in the SSc-ILD group and gradually increased in the SSc and control groups. Chen et al^18^ found similar results to ours in their study of RA patients with and without ILD. Although lymphopenia may occur in various autoimmune diseases due to the use of steroids and immunosuppressants, the patients in the study were not receiving steroid treatment. Furthermore, there was no difference in lymphocyte counts between patients receiving immunosuppressants and those taking only HQ. Therefore, changes in PLR, NLR, and LMR may be considered to reflect disease activity. In this study, a moderate positive correlation was found between PLR and NLR and CRP.

Recently, clinicians have increasingly turned to new inflammation-based indices such as SII, SIRI, and PIV, in addition to NLR and PLR, to assess disease activity and prognostic indicators in different types of inflammatory conditions. These novel inflammation markers seem to offer greater stability than individual blood count values and can be computed within minutes.^20^

Determining disease activity in connective tissue diseases is difficult. This evaluation requires a long time and is costly in outpatient clinic conditions, especially in the group with visceral organ involvement. Yang et al^21^ observed that SII and SIRI values were markedly elevated in patients with SLE compared to the control group. Additionally, they found that SII and SIRI values were significantly elevated in patients with nephritic involvement compared to those without nephritis, concluding that these values can be used as biomarkers to evaluate and predict the occurrence of SLE, disease activity, and lupus nephritis.

In line with the study, Okutan et al’s^6^ research on RA patients demonstrated that SII, SIRI, and PIV values were significantly elevated in the patient group compared to the control group. A study by Zinellu et al., involving 73 patients with IPF and 62 healthy controls, found that NLR, LMR, SIRI, and PIV were associated with IPF.^22^

To ascertain the efficacy of these markers in indicating activity in SSc patients, it was observed that SII, SIRI, and PIV values were markedly elevated in the patient group. The SIRI was identified as the most effective marker, exhibiting 66% sensitivity and 66.7% specificity, with a cutoff value of 0.89. Similarly, when assessing SSc patients with ILD, it was determined that the most effective marker was SII, demonstrating a sensitivity of 75% and specificity of 74.7%, with a cutoff value of 642.97. In a study conducted by Okutan and colleagues on patients with RA, the researchers confirmed that ROC analysis could indicate active RA if PIV, SII, and SIRI values were above certain thresholds.^6^

The findings of the present study show that SII, SIRI, and PIV are elevated in patients with SSc compared to the control group and higher in patients with ILD compared to those without. While CRP elevation is considered an indicator of disease activation, an increase in these parameters is also associated with disease activation. Correlation analysis revealed a moderate positive correlation between these parameters and CRP. These results suggest that new indexes could be useful for predicting disease activation and IPF formation in patients with SSc.

It is imperative to acknowledge the limitations of this study when interpreting the findings. The primary constraint pertains to the relatively modest sample size, which may not be representative of the broader population and could potentially compromise the statistical power to discern specific trends or relationships. Secondly, the study’s retrospective design constitutes a limitation. One additional limitation of the study is that it was conducted at a single center, which raises questions about the generalizability of the results to other settings or populations. Given the retrospective nature of the study’s design, it is important to highlight that a comparison of patients’ lung function was not possible. This was because the researchers did not have access to the patients’ pulmonary function tests.

The SII, SIRI, and PIV were found to be elevated in patients with SSc compared to the control group and in patients with IAH compared to those without IAH. These novel indices under discussion in the studies may prove superior to rudimentary indices by virtue of their capacity to more accurately reflect inflammatory activity in inflammatory connective tissue diseases, particularly those accompanied by fibrotic lung involvement. Moreover, their simplicity and cost-effectiveness increase the utility of these indices and make them important for early diagnosis and assessment of disease activity in SSc. However, large-scale, prospective studies are now needed to investigate their prognostic role in SSc.

## Figures and Tables

**Table 1.** Characteristics of Patients and Controls, Including Clinical and Demographic Data

	Patients (n = 53)	Controls (n = 54)	*P*
Age	53.32 ± 13.29	49.74 ± 8.36	.098
Sex (male/female)	4/49	4/50	.978
Clinical manifestations (n)			**%**
Skin	43		81
Raynaud’s phenomenon	33		62
Articular	28		53
Gastrointestinal	4		7,5
Interstitial lung disease	24		45
Pulmonary arterial hypertension	8		15
Medications (n)			
Only hydroxychloroquine	15		28
Only Immunosuppressive (mycophenolate mofetil or azathioprine)	10		19
Hydroxychloroquine + immunosuppressive (mycophenolate mofetil or azathioprine)	28		53

**Table 2.** Comparison of Laboratory Findings Between the Patient and Control Group

	Patients (n = 53)	Controls (n = 54)	*P*
	Mean ± Standard deviation	

White blood cell (10^3^ μL)	7.01 ± 1.78	7.22 ± 1.37	.49
Lymphocyte (10^3^ μL)	1.96 ± 0.71	2.36 ± 0.55	**.001**
Monocyte (10^3^ μL)	0.46 ± 0.17	0.44 ± 0.13	.384
Platelet (10^3^ μL)	281.19 ± 87.87	274.78 ± 53.49	.649
	Median (min-max)	

Neutrophil (10^3^ μL)	4.20 (1.64-7.85)	4.02 (2.53-7.61)	.616
ESR (mm/hour)	10.50 (2-52)	9 (0-41)	.916
CRP (mg/L)	1.9 (0.30-15)	1.89 (0.10-8)	.205
NLR	2.32 (1.09-8.80)	1.69 (0.90-4.08)	**.001**
PLR	136.60 (51.08-450.70)	122.20 (50.86-195.60)	**.01**
LMR	4.19 (1.09-46.33)	5.89 (2.19-32.86)	**<.001**
SII	645.89 (125.65-2312.10)	467.63 (193.92-1181.82)	**.012**
SIRI	1 (0.05-3.67)	0.77 (0.10-2.77)	**.003**
PIV	265.41 (3.79-1156.05)	183.12 (17.85-776.93)	**.013**

Bold values are statistically significant.

CRP, C-reactive protein; ESR, erythrocyte sedimentation rate; LMR, lymphocyte-to-monocyte ratio; NLR, neutrophil-to-lymphocyte ratio; PIV, pan-immune-inflammation value; PLR, platelet lymphocyte ratio; SII, systemic inflammatory index; SIRI, systemic inflammatory response index.

*The Mann–Whitney *U-*test was performed for NLR, PLR, LMR, SII, SIRI, PIV, ESR, CRP, and neutrophil count. The Student *t*-test was performed for the others

**Table 3.** Comparison of Laboratory Findings Between the Patient with and Without ILD and Control Group

	Patients with ILD (n = 24) (a)	Patients without ILD (n = 29) (b)	Controls (n = 54)(c)	*P* (a-b)	*P* (b-c)	*P* (a-c)
	Mean ± Standard deviation					
Age (years)	57.46 ± 12.80	49.90 ± 12.90	49.74 ± 8.36	.38	.947	**.002**
Sex (F/M)						
White blood cell (10^3^ μL)	6.99 ± 1.74	7.03 ± 1.84	7.22 ± 1.37	.927	.592	.519
Lymphocyte (10^3^ μL)	1.79 ± 0.69	2.1 ± 0.70	2.36 ± 0.55	.111	.065	**<.001**
Monocyte (10^3^ μL)	0.46 ± 0.14	0.46 ± 0.19	0.44 ± 0.13	.996	.476	.444
Platelet (10^3^ μL)	302.71 ± 69.84	263.38 ± 98.02	274.78 ± 53.49	.105	.494	.057
	Median (min-max)					
Neutrophil (10^3^ μL)	4.34 (2.37-7.85)	4.02 (1.64-6.43)	3.99 (2.53-7.61)	.611	.863	.499
ESR (mm/hour)	12 (3-52)	8 (2-40)	9 (0-41)	.098	.472	.305
CRP (mg/L)	2.75 (0.70-15)	1.4 (0.30-11.2)	1.87 (0.10-8)	**.016**	.731	**.009**
NLR	2.66 (1.16-8.80)	1.95 (1.09-3.43)	1.67 (0.90-3.64)	**.028**	.065	**.001**
PLR	170.29 (89.37-450.70)	115.88 (51.08-311.63)	122 (50.86-195.60)	**.001**	.811	**<.001**
LMR	4.13 (1.09-9.70)	4.35 (1.95-46.33)	5.47 (3.14-32.86)	.163	**.007**	**<.001**
SII	806 (297.41-2312.10)	465.85 (125.65-1788.74)	458.94 (193.92-1181.82)	**.006**	.626	**<.001**
SIRI	1.14 (0.41-3.67)	0.97 (0.05-1.87)	0.76 (0.10-2.04)	.148	**.047**	**.004**
PIV	324.98 (105.28-1156.09)	227.29 (3.79-1001.70)	182.71 (17.85-661.82)	**.035**	.344	**.001**

Bold values are statistically significant.

CRP, C-reactive protein; ESR, erythrocyte sedimentation rate; LMR, lymphocyte-to-monocyte ratio; NLR, neutrophil-to-lymphocyte ratio; PIV, pan-immune-inflammation value; PLR, platelet lymphocyte ratio; SII, systemic inflammatory index; SIRI, systemic inflammatory response index.

**Table 4.** Comparison of Laboratory Findings Due to Medicine

	IMMSUP+HQ (n = 28) (a)	HQ (n = 15) (b)	IMMSUP (n = 10) (c)	*P* (a-b)	*P* (b-c)	*P* (a-c)
	Mean ± Standard deviation					
Age (years)	52.68 ± 14.55	54.93 ± 11.44	52.70 ± 13.23	.606	.997	.677
White blood cell (10^3^ μL)	7.00 ± 1.66	6.86 ± 1.26	7.27 ± 2.92	.779	.713	.615
Lymphocyte (10^3^ μL)	1.92 ± 0.74	2.00 ± 0.60	1.98 ± 0.83	.698	.826	.932
Monocyte (10^3^ μL)	0.46 ± 0.19	0.45 ± 0.11	0.51 ± 0.18	.847	.428	.279
Platelet (10^3^ μL)	290.18 ± 95.78	253.87 ± 76.51	297.00 ± 78.83	.213	.841	.186
	Median (min-max)					
Neutrophil (10^3^ μL)	4.34 (2.37-7.85)	4.02 (1.64-6.43)	3.99 (2.53-7.61)	.610	.987	.978
ESR (mm/hour)	12 (3-52)	8 (2-40)	9 (0-41)	.211	.960	.461
CRP (mg/L)	2.75 (0.70-15)	1.4 (0.30-11.2)	1.87 (0.10-8)	.256	.442	.129
NLR	2.66 (1.16-8.80)	1.95 (1.09-3.43)	1.67 (0.90-.,64)	.212	.832	.531
PLR	170.29 (89.37-450.70)	115.88 (51.08-311.63)	122 (50.86-195.60)	.702	.961	.196
LMR	4.13 (1.09-9.70)	4.35 (1.95-46.33)	5.47 (3.14-32.86)	.319	.987	.643
SII	806 (297.41-2312.10)	465.85 (125.65-1788.74)	458.94 (193.92-1181.82)	.231	.935	.367
SIRI	1.14 (0.41-3.67)	0.97 (0.05-1.87)	0.76 (0.10-2.04)	.491	.636	.311
PIV	324.98 (105.28-1156.09)	227.29 (3.79-1001.70)	182.71 (17.85-661.82)	.114	.757	.261

CRP, C-reactive protein; ESR, erythrocyte sedimentation rate; LMR, lymphocyte-to-monocyte ratio; NLR, neutrophil-to-lymphocyte ratio; PIV, pan-immune-inflammation value; PLR, platelet lymphocyte ratio; SII, systemic inflammatory index; SIRI, systemic inflammatory response index.

**Table 5.** ROC Analysis Results of SII, PIV, and SIRI Parameters for Systemic Sclerosis (a)/Systemic Sclerosis with ILD (b)

	Cutoff	*P*	AUC	Sensitivity (%)	Specificity (%)
SII (a) SII (b)	533.7 642.97	.12 **<.001**	0.642 (0.535-0.748) 0.756 (0.636-0.875)	58.5 75	61.1 74.7
SIRI (a) SIRI (b)	0.89 0.96	**.003** **.01**	0.665 (0.561-0.769) 0.673 (0.538-0.809)	66 62.5	66.7 63.9
PIV (a) PIV (b)	228.07 253.36	**.013** **.002**	0.640 (0.533-0.747) 0.710 (0.588-0.833)	60,4 66.7	61.1 65.1

Bold values are statistically significant.

PIV, pan-immune-inflammation value; SII, systemic immune-inflammation index; SIRI, systemic inflammation response index.

**Table 6.** Relationships Between Blood Cell Count Indexes and Classical Activity Parameters

		ESR	CRP
SII	*r*	0.422	0.444
	*P*	**0.002**	**0.001**
SIRI	*r*	0.291	0.323
	*P*	**0.036**	**0.018**
PIV	*r*	0.383	0.432
	*P*	**0.005**	**0.001**
NLR	*r*	0.29	0.338
	*P*	0.772	**0.000**
PLR	*r*	0.148	0.356
	*P*	0.132	**0.000**
LMR	*r*	−0.036	−0.093
	*P*	0.715	0.341

Bold values are statistically significant.

CRP, C-reactive protein; ESR, erythrocyte sedimentation rate; LMR, lymphocyte-to-monocyte ratio; NLR, neutrophil-to-lymphocyte ratio; PIV, pan-immune-inflammation value; PLR, platelet lymphocyte ratio; SII, systemic inflammatory index; SIRI, systemic inflammatory response index.

## Data Availability

The data that support the findings of this study are available on request from the corresponding author.
